# Social network participation towards enactment of self‐care in people with chronic obstructive pulmonary disease: A qualitative meta‐ethnography

**DOI:** 10.1111/hex.13340

**Published:** 2021-08-25

**Authors:** Lindsay Welch, Euan Sadler, Anthony Austin, Anne Rogers

**Affiliations:** ^1^ School of Health Sciences, Faculty of Environmental and life sciences University of Southampton Southampton UK; ^2^ Patient and Public Involvement Group Representative, Long Term Conditions PPI group University Hospital Southampton NHS Foundation Trust Southampton UK

**Keywords:** chronic obstructive pulmonary disease, long‐term conditions, management, qualitative meta‐ethnography, self‐care, self‐management engagement, social network participation

## Abstract

**Background:**

How people with chronic obstructive pulmonary disease (COPD) engage with supportive social networks to enhance self‐care is not understood. The personal rationales for participation in socially directed support have not been addressed in the literature. To determine how people with COPD identify, engage and participate in socially supportive self‐care practices, we conducted a systematic review and meta‐ethnography of qualitative studies.

**Methods:**

A systematic literature search was conducted between June 2010 and June 2021. Of 3536 articles, 8 fulfilled the inclusion criteria. Using a meta‐ethnography approach to the qualitative synthesis, new concepts were derived from the data to identify aligning themes and develop a conceptual model.

**Findings:**

Interpretations from the papers yielded concepts of (1) accountability and personal responsibility in self‐care, (2) valued positive relationships with clinicians, (3) understanding of illness through shared and personal experiences and (4) acknowledging social networks in fostering self‐care engagement in people with COPD. The independence‐experience (Index) model of synthesized (third order) interpretations highlighted the processes of social networks and self‐care practices: (a) fear or avoidance of dependency, (b) learning from experiences of adaptive self‐care behaviours and (c) including valued practices in self‐care. Self‐care strategies are formed through illness experiences and relatable social encounters.

**Conclusion:**

The model derived from the third‐order interpretations is a framework to describe socially supported self‐care and can be used to direct future self‐care strategies and target interventions for people with COPD.

**Patient or Public Contribution:**

The findings and model were presented to the long‐term conditions patient and public involvement group. The manuscript is coauthored by a public representative.

## 1 BACKGROUND AND RATIONALE

Self‐management interventions^1^, ^2^, ^3^ for people with chronic obstructive pulmonary disease (COPD) have been found to contribute towards reducing the burden of disease, improving quality of life and reducing the risk of hospitalisation.^4^ Challenges to enacting self‐care in COPD include low health literacy,^5^ multi‐morbid disease^6^ and low self‐efficacy^7^ in understanding and managing complex, sometimes frightening symptoms.^8^ Supportive social networks are recognized as relevant for accessing social resources and enhancing individual capabilities for chronic illness self‐management.^9^ Social support is a valuable coping mechanism for people with COPD, associated with reduced hospitalisations and fewer exacerbations, and in the general population, insufficient social connections are linked to higher mortality.^3^, ^10^, ^11^, ^12^ Participation in social support networks enables individuals with COPD to embed self‐care practices into their personal everyday lives.^3^, ^13^ However, this is currently underexplored in the context of COPD.^3^, ^14^ There is a need to understand and explain the value of socially supported self‐management in people with COPD^14^, ^15^ to inform integrated care pathways and community interventions aimed at increasing self‐care practices in this population.^16^

Evidence suggests that psychosocial aspects such as addressing anxieties and family dynamics are relevant to supporting behavioural change in people with COPD,^17^, ^18^ but are not routinely included in self‐management consultations with healthcare professionals.^14^, ^19^ Furthermore, evidence suggests that accessing community resources and support from a wide range of social network support^20^ enables the maintenance of positive health behaviours and reduces unnecessary contact with health professionals.^21^, ^22^ Peer and social network support offers potential support in personal decision making and the maintenance of longer‐term health‐related activities.^23^, ^24^ This review examines how social network participation enables self‐care engagement among people with COPD to inform the development of future social network interventions for this population.

### 1.1 Aim (research question)

The aim of this study was to understand the personal and contextual influences of how social self‐management support (SSMS) practices are selected and established in the everyday lives of people with COPD.

### 1.2 Objective(s)

The objectives of this study were to investigate how people with COPD identify, engage and select socially supportive self‐care resources and to explore the process of adoption of socially supportive self‐care practices in people with COPD.

## 2 METHODS

A systematic review and meta‐ethnography of qualitative studies was undertaken,^25^ using the seven‐step method of meta‐ethnography, as described by Noblit and Dwight Hare^26^, ^27^ and exemplified by Sinnott et al.^28^

In Step 1, we developed a specific research question and its contribution to the field.

In Step 2, a search strategy was devised to ensure that the studies selected would address the research question. The focus of the systematic review and qualitative synthesis was to develop an in‐depth understanding of the phenomenon of socially supportive self‐management among people with COPD. Search terms were designed to ensure that the qualitative studies selected examined how people with COPD engaged with socially supportive self‐management practices. Seven databases were searched: CINAHL, MEDLINE (OVID), APA PsycInfo, Web of Science, PubMed, Cochrane Library and EMBASE. This was supplemented by citation tracking and grey literature searches. The search was limited to English‐language papers between June 2010 and June 2021. The search terms used were COPD (and synonyms) AND Chronic Obstructive Pulmonary Disease OR COPD OR Emphysema AND Self‐management OR self‐care OR self‐management support OR social network support AND qualitative studi.

**Figure 1:**
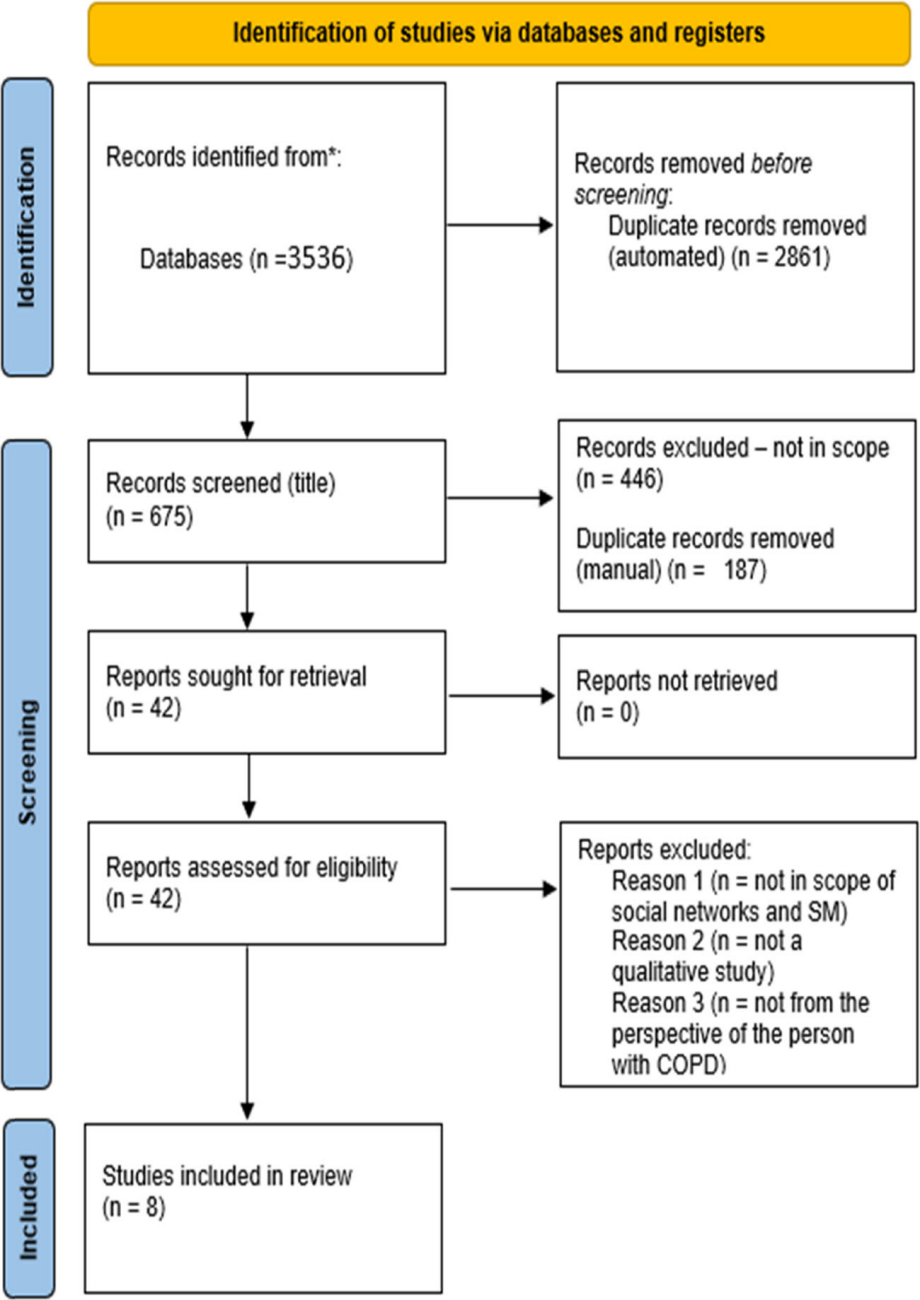
Prisma flow diagram of literature review and selection. COPD, chronic obstructive pulmonary disease

Initial titles and abstracts of papers were read by one reviewer (L. W). Full articles of potentially eligible studies were reviewed by two researchers (L. W. and E. S). Inclusion criteria focused on qualitative studies that explored the enactment of socially supported self‐management in people with COPD. The quality of the studies included was assessed using a quality appraisal tool developed by the British Sociological Association.^29^ This tool has been widely used in similar reviews.^30^, ^31^ Quality was not a criterion to exclude studies, but instead provided critical insights into the methods used for data collection and analysis.^28^ The selection process of studies is presented in the PRISMA flow diagram (Figure 1).^32^

Selected studies aligned to the research question on the basis of detailed inclusion and exclusion criteria (Table 1).

**Table 1:** Inclusion and exclusion criteria

Inclusion criteria	Rationale
Qualitative studies focused on personal narratives and the perspectives of people with COPD	The aim is to integrate and reinterpret qualitative findings
Studies discussing people with COPD	To provide specificity of the long‐term condition and the nature of the types of interventional studies
Studies addressing the broad concepts of socially supportive social networks in COPD self‐management or engagement and social participation in network support	To understand participation in social supportive self‐care or self‐management support
Fulfils quality criteria^29^	Guidelines for quality appraisal to ensure that the methods and analysis of the selected studies were rigorous.
Exclusion	Rationale
Non‐English Language	Unable to interpret or integrate
Focused on a nuanced area of COPD care delivery (pulmonary rehabilitation or oxygen therapy)	Interventional specificity
Focused on a single self‐management intervention (a specific eHealth programme)	Interventional specificity
Not disease‐specific	Not specific to the investigational population
If the studies are from the perspective of health professionals or carers alone	Not a personal narrative from a person with COPD

Abbreviation: COPD, chronic obstructive pulmonary disease.

In Step 3, selected studies were read and reread by two authors (L. W. and E. S). Study findings were entered into an extraction table (Table 2). This included both first‐order interpretations (views of the participants) and second‐order interpretations (views of the authors; Table 3).

**Table 2:** Data extraction table

Methods and concepts	Slevin et al.^33^	Sheridan et al.^34^	Apps et al.^35^	Fotokion et al.^36^	Franklin^37^	Zeb et al.^38^	Glenister et al.^39^	Willard‐Grace et al.^40^
Sample	Convenience sample, a selection of 30 COPD patients with no life limiting comorbidities. *N* = 30	Pragmatic sampling of 2 groups: Rural and poor urban. Grouped for ethnicity, all of whom have had two or more admissions due to COPD in the last 12 months. Group 1 = 13, Group 2 = 21, *n* = 33	Patients with mild‐ moderate COPD in primary care. An opportunistic sample. *N* = 15	An Iranian criterion‐based and purposive sampling of people with COPD over 60 years of age, their family care giving or HCP. They had to have the ability to explain their experiences	Literature review of six databases using Boolean logic from 2004 to 2010. 5106 articles CASP reviewed. *N* = 14	13 People with COPD, purposeful sampling in an outpatient clinic in Northern Pakistan, of lower socioeconomic status and in joint families	A convenience sample of 14 people with COPD consented to interview, recruited from a subregional Australian hospital	36 Participants, low‐income, minority population with psychiatric conditions and substance use, and household instability
Data collection	Semi‐structured interviews and in‐depth questionnaires with open‐ended questions to ascertain use of DHT for SMS	In‐depth exploratory interviews conducted in the home, using an interview topic guide in the native or preferred language	Face‐to‐face semi‐structured interviews (nested in a larger quant SMS study). Using the Braun and Clarke framework	Grounded theory, in‐depth semistructured interviews	PRISMA statement used to guide qualitative synthesis	Face‐to face semistructured interviews conducted over 2 months in 2019	Qualitative semi‐structured interviews. Thematic analysis, to remain aligned to the data and not overanalyse the text	In‐depth semi‐structured interviews with participants, clinicians and health coaches
					Thematic synthesis and interpretivist approach to analysis. Thomas and Harden 3 principles for thematic synthesis	Interpretive phenomenological analysis used to capture the detail of the lived experience		
Overarching concept or study purpose	COPD patients' perceptions of the benefits of DHT and how this will support their SM	Understanding the experience of living with COPD in differing ethnic populations. Managing symptoms and therapies, self‐care and receiving healthcare. Understanding the ability to self‐manage	Experiences of dyspnoea, personal perceptions of COPD and the expectations of understanding of self‐management strategies	How people act and respond to problems that they encounter in SMS. An exploration of COPD elder empowerment as an interactional concept	The aim was to deepen the understanding of self‐management, and the aim of people was to maintain a ‘normal’ life through perceptions and experience of patients	The aim is to explore the role of the family in self‐care in people with COPD	To understand the experience of COPD and social connectedness in a rural context. An understanding in ageing‐in‐place with COPD	Understand the effectiveness of lay health coaching to meet the needs of vulnerable people with COPD. Health coaches MI‐trained
	Patient perceptions of disease management with DHT and treatment							
Experiencing and understanding the disease and symptoms	Patients believe that DHT monitoring will support their decision making around accessing healthcare resources	Reports of struggling to live with debilitating symptoms of breathlessness and fatigue. Diagnostic uncertainty and an uncertain trajectory	Adapting to symptoms, diagnostic uncertainty and uncertainty of progression, unsure how to manage exacerbations or exercise	Striving to keep abreast of life by information‐seeking from peers and nonprofessionals	Patients reported knowing that they have responsibility, but finding it too hard to put into practice. Expectations were too high	The disease is a curse from god, and prayer can be a weapon against it	Learning to cope with an illness and adaptations to a new normal.	Lay explanations of disease and support with aspects of a person's life that they value the most
					Diagnostic and disease progression uncertainty	Western medicine is unaffordable and traditional remedies are used	Learning when to seek help	
Accountability, responsibility and blame	DHT could foster ‘self‐efficacy’, increasing the confidence in the ability to take on and manage tasks associated with the disease	Feelings of helplessness, letting go, going with the symptoms	Issues with the redistribution of everyday tasks or usual family work. Overall, though, a lack of symptom control was felt	Extrinsic societal influences can enhance or destabilize an elderly patient's life. Often, elders undertake poor self‐treatment or incorrect self‐treatment	People would prefer to balance illness and work with existing habits.	Self‐care is a privilege for the rich. Avoiding triggers and aggravating symptoms often were at odds with warmth and food—basic needs	Not addressed	Managing illness and changing health behaviours are internalized
		Self‐blame in the European participants reinforced by guilt and shame			The need to make the ‘right choices’—requires discipline, but have self‐blame and guilt			
Participating in care	DHT has the potential to optimize the consultation experience by empowering people to participate in collaborative conversations using their DHT recordings	Participants had no recollection of SMS strategies and no understanding of early symptom recognition. Conflicting information provided by HCP; however, people valued established relationships with HCP	Not addressed	Often, they are preparing to ‘do battle with disease’ and to cooperate with HCP and family. This requires trust in HCP	Challenges in the practical application of knowledge. Peopled valued being listened to along with consideration of individual circumstances	Priorities of faith, cultural and traditional approaches to self‐*care* were valued over medical and nursing advice and management	Knowing and building relationships with doctors and GPs. Positive relationships, and open, candid conversations were valued	Enhanced participation in care, through lay support
								Enabling participation through supportive negotiation of services. Health coaches are not doctors, less judgemental
Psychosocial needs in SMS	Patients perceive that DHT could reduce feelings of anxiety associated with their COPD as they monitor symptoms and can access advice	Faith in god, the church and family were the most valued activities in the Pacific islander groups. These came before disease and health	Success in the redistribution of work and tasks can help to aid SMS in COPD; however, this can reduce social role, positioning and importance	Peer information‐seeking; not always correct information is often valued. Nurses are viewed as translators of care, especially for illiterate people	Patients preferred to discuss psychosocial issues and personal experiences. Linear scoring disease in terms of management by HCP increased anxiety, not compliance	Reciprocal family priority supported people with the burden of their disease and in their self‐care. Therefore, the emotional, financial and physical support with the disease was selflessly absorbed by the family. Distant and close relatives genuinely made themselves available to provide care emotionally and physically	Loss of social identity and loss of social role (job loss)	Focus on psycho‐social needs. Housing and environment
				A wish to reduce dependencies				Relational aspects of care most valued
Main findings or theory (second‐order construct)	DHT improves the capacity and understanding to respond to symptom changes, i.e., exacerbations of COPD, and prompts patients to make proactive decisions regarding their treatment	Differences arise between the European and Pacific Islander groups. Negative attitudes towards SMS due to self‐blame and social isolation; these were conversely positive in the Pacific islanders due to associations with the church and the value of family	Independent initiation of self‐care behaviours through experience with no formal support. Unaware that this is SMS, and often, participants lacked confidence in initiating formal clinical management of COPD	Knowing—The knowing that is derived from experiential constructs can both positively and negatively influence care participation decisions	Dominant finding = the dominance of the traditional model of care in the context of individual responsibility and accountability. Overarching reporting structures direct care away from truly patient‐centred approaches. Patients value a broader set of social influences that shape behaviours	The priority is family and children, in a resources poor setting they are the priority and care is provided by the family	The importance of inclusion rather than isolation for disease support	Value of relationship building and trusting relationship in lay supporters is highly beneficial for people with COPD
							Value of the ‘inclusive village’	
							Community support (bus drivers and others) supporting to maintain independence	
Third‐order constructs—developing a line of argument	DHT validates complex symptoms; this validation reduces anxiety. *The validation of symptoms* then encourages help‐seeking, as people feel that they have evidence (as in peer discussion)	Helplessness feeds a poor perception of disease control. *Valued activities* and *social groups*, above those of disease control, can positively enhance coping and disease management in COPD	Evolution of self‐care through experiential learning and working through *previous illness experiences*	Attribution of knowing and building a personal disease skill set. Learning through *experience* and induction	*The temporal nature of interactions* with HCP. Participants ceased interactions if they felt that they were not valued	Family support within the nuclear family and within the wider family was encouraging and motivating	Importance of work–life balance to maintain independence and face *sociocultural illness challenges*	Relationships and valued opinions of peers and lay support
	Furthermore this ‘evidence of symptoms’ supports collaborative conversations and levels of healthcare consultations				*Valued experiential strategies* including *wider social influences* to shape learning and share peer knowledge and also to continue to reduce anxiety around disease control			

Abbreviations: COPD, chronic obstructive pulmonary disease; HCP, health care professional.

**Table 3:** Analytical framework

Metasynthesis	Ontological positioning	Inclusion	Framework analysis	Indexing	Output
Integrative and interpretive	Interpretivist	All qualitative studies included	Codes clustered around new ideas	Reapplication of the codes	New themes or concepts
		Predefined research question			

In Step 4, we determined how the studies were related to each other by comparing individual study findings. The ontological stance of subjectivism, or interpretivism, was selected.^41^

In Step 5, studies were ‘translated into each other’, which involved examining the contribution of each study to a key theme or concept. Each theme or concept emerged from individual studies, but was also viewed as relevant to the studies included in the synthesis. The process of data extraction and linking of the concepts is presented in Table 4.

**Table 4:** Translation of the key concepts through the studies—postdata extraction

Second‐order interpretations	Evidence in the paper	Evidence in the paper	Evidence in the paper	Evidence in the paper	Evidence in the paper	Evidence in the paper	Evidence in the paper	Evidence in the paper
	Paper 1 Slevin et al.^33^	Paper 2 Sheridan et al.^34^	Paper 3 Apps et al.^35^	Paper 4 Fotokion et al.^36^	Paper 5 Franklin et al.^37^	Paper 6 Zeb et al.^38^	Paper 7 Glenister et al.^39^	Paper 8 Willard‐Grace et al.^40^
Balancing social network participation with self‐care accountability and personal responsibility	Digital health technology (DHT) fosters self‐efficacy and independence. Increases confidence in completing SMS tasks associated with COPD	Helplessness undermines a personal ability to engage in SM	People with COPD reported being unsure of what constitutes an SM activity	Independence seeking; older people with COPD seek to reduce dependencies on others	An assumed responsibility and accountability for making the right care or treatment choices	People balance self‐care with finances and family. Often accountable for their care, but choosing to put family first	Understanding the experiences of COPD and social connectedness in a rural context	Managing illness and changing health behaviours can be internalized
		Often leading to devising personal management strategies						Challenging to discuss with professionals
The value of positive engagements with healthcare professionals where socially supported self‐care is relevant	DHT promotes an equal discussion with health professionals. DHT records evidence of symptoms and supports articulation of symptoms in consultations	Frustration over conflicting information from health professionals (issues with negative consultations)	HP can support people with COPD to gain the maximum benefit from their SM endeavours	People engage in care processes only with trusted healthcare providers	People reported that generic education was not relatable. People wanted strategies to apply knowledge to individual situations	Access to formal care provision is at a cost. Relationships are with informal healthcare providers and lay healers	Positive relationships and open, candid conversations were valued with local rural healthcare professionals	Lay coaching bridges this relationship. Aids service negotiation and honest conversations
				People would value recognition of personal health status, mood and issues	‘Unheard’ patients reduced SM			
Developing a personal understanding of illness through social participation and shared and personal experiences	DHT prompts personal proactive responses to symptom changes	Decisions shaped by experiences of failure in SM	Strength loss and fatigue not associated with COPD	External information‐seeking through peers. This information was deemed more accessible	People reported having poor understanding of what constituted healthy and unhealthy choices	The family, spirituality and community are highly valued. Some health beliefs are culturally nuanced, such as the belief that disease is a curse from god	Learning to cope with and balance social life and adapt to new illness symptoms, learning when to seek help and link with others	Lay explanations of disease and support with aspects of a person's life that they value the most
		COPD confused with asthma, so misleading illness trajectories	Trial and error adaptations to daily living were most acceptable	Knowing that is derived from experiential constructs				
Recognizing the importance of social networks to guide and validate personal choices in people with COPD	Reassurance of support through online/offline feedback	God, church and the family valued above all other things	Poor social networks lead to frustration, unable to link with others. Positive networks foster discussions to adapt tasks and SM with other network members	Familial groups can empower people by providing communication channels to the outside world	The behaviours and choices of people with COPD were shaped by a broader social context	Self‐care is encouraged and delivered by the extended family, including emotional and social needs. It is a selfless act, valued and encouraged	Learning when to seek help, from the community through a community infrastructure	Focus on psychosocial needs, housing and environment
	Although not a person, people valued discussion around their condition	Social isolation adds to the emotional burden						Relational aspects of care most valued
		Cultural value of social networks, people living alone struggled with SM						

Abbreviation: COPD, chronic obstructive pulmonary disease.

Step 6 involved generating third‐order interpretations from the main findings of the synthesis. These are new concepts directly derived from the interpretation of second‐order concepts (Table 4). To demonstrate this process, an extraction table was used to collate and analyse the qualitative data. This created a visual breakdown of concepts from the studies included, enabling the synthesis of linked concepts across studies^27^ (Tables 2 and 4). A line of argument synthesis^27^, ^31^ (synthesis refinement) linked common concepts together into new theory, in turn demonstrating that the studies agreed with each other and could be translated back into one another (Table 4).

Finally, Step 7 reports the synthesis. The Supporting Information Tables and the discussion present the stages of synthesis and findings, which were further depicted through the development of conceptual models to illustrate interactions of the phenomenon examined (Tables 3 and 4, Models 2A,B).

## 3 RESULTS

Search outcomes are presented in the PRISMA flow diagram (Figure 1). The searches retrieved 3536 studies, 675 (after removal of duplicates); 42 studies were screened at the full‐text stage, of which eight papers were eligible for inclusion in the review.

Qualitative studies were included in the review if they addressed the broad concepts of socially supportive social networks in COPD self‐management, engagement and social participation in network support and examined participation through the lens of a person with COPD. Studies were excluded if they focused on a single self‐management intervention (a specific eHealth programme), focused on a specific medical treatment of COPD care delivery (e.g., pulmonary rehabilitation or oxygen therapy), perspectives of family carers or health professionals or were not reported in the English language.

Eight studies were included in this review. Slevin et al.,^33^ Sheridan et al.,^34^ Apps et al.,^35^ Fotokian et al.,^36^ Franklin et al.,^37^ Glenister et al.,^39^ Zeb et al.^38^ and Willard‐Grace et al.^40^ reported enactment and engagement in supportive social networks for illness management of COPD using a range of approaches. These were digital health technology (DHT) for symptom assessment and to aid symptom negotiation; experiences of enacting social network support for people from diverse cultural backgrounds; personal perceptions of COPD; managing symptoms of COPD including dyspnoea; and problem solving in older people with COPD and their networks and understanding how people maintain a normal life with disease using social network support. Studies included descriptions of social interventions, including lay health coaching for vulnerable people with COPD, and family support networks.

Studies were conducted in Australia,^39^ New Zealand and the Pacific Islands,^34^ the United States of America,^40^ Iran^36^ and Pakistan,^38^ and the United Kingdom.^33^, ^35^, ^37^

**Figure 2:**
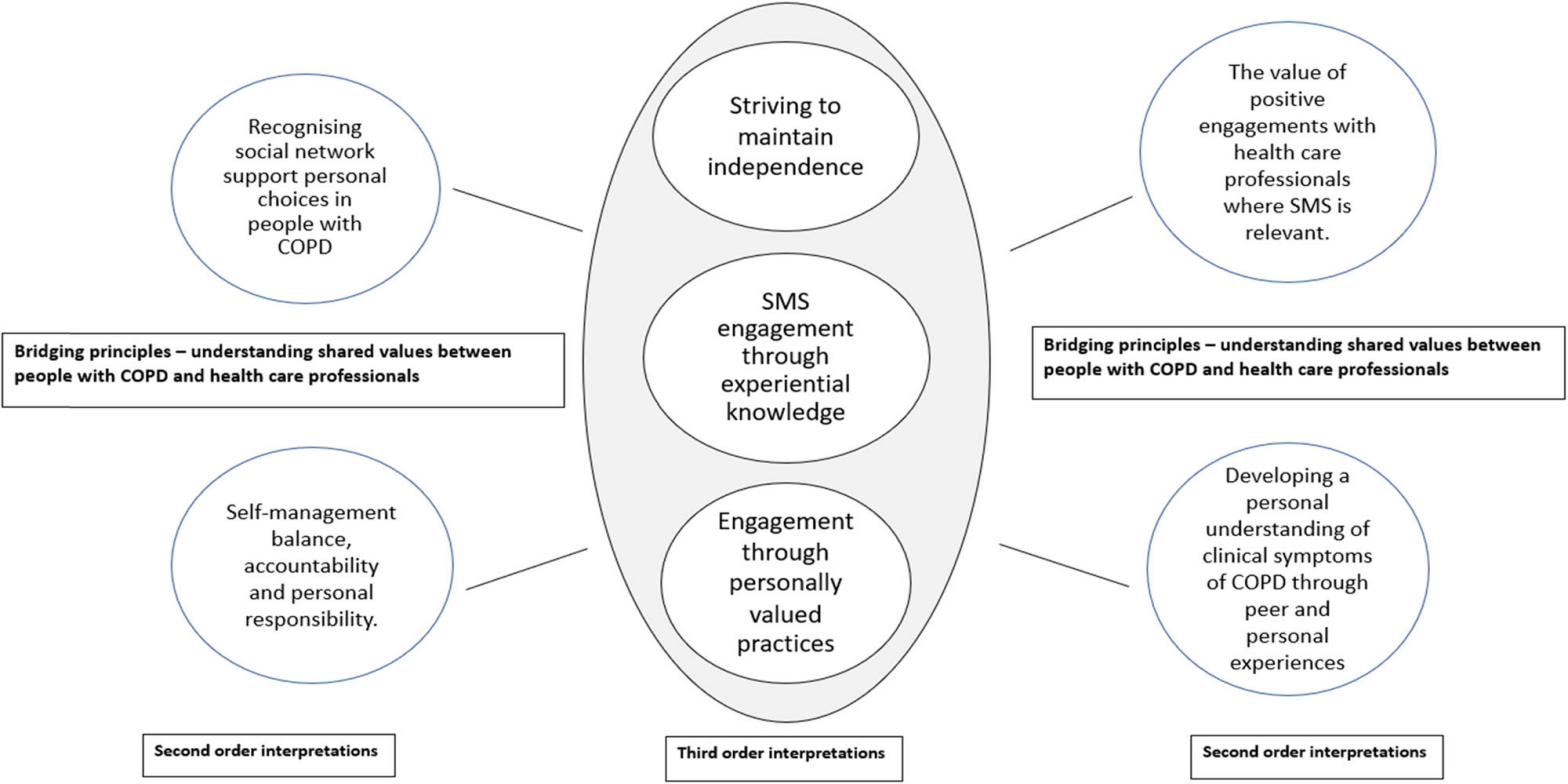
Principles of enactment and engagement. Further synthesis of second‐order to third‐order themes. COPD, chronic obstructive pulmonary disease

Key characteristics of the included studies (e.g., sample, methods and findings) were added to a data extraction table (Tables 2 and 4). A concept map, Figure 2, is presented to illustrate the alignments and links of the concepts to justify interpretations. The concept map forms the basis of a conceptual model of engagement and enactment of SSMS in people with COPD.

### 3.1 Synthesized findings

This meta‐ethnography generated four second‐order interpretations:

*Balancing social network participation with self‐care accountability and personal responsibility*.

*The value of positive engagements with healthcare professionals where socially supported self‐care is relevant*.

*Developing a personal understanding of illness through social participation, shared and personal experiences*.

*Recognizing social network support to guide and validate personal choices for people with COPD*.

These concepts suggest that enactment of self‐care practices and social engagement is perceived as challenging due to the everyday uncertainty of the disease and previous negative illness experiences. Individuals living with COPD who were confident in the knowledge of their illness and interpretation of illness symptoms felt more able to engage with supportive social networks, compared to those who had not yet been able to comprehend their own illness experiences or the impact of their symptoms on their daily lives.

1. Balancing social network participation with self‐care accountability and personal responsibility.
  The progression of COPD and mobility limitations caused by increasing breathlessness creates the need to redistribute everyday work, heavy work and family responsibility.^35^ People with COPD reported that they would like to balance their illness work with existing habits and understood that they needed to make the ‘right’ choices in terms of lifestyle and self‐care. However, the challenges to have the discipline to do this created a perceived conflict of personal accountability for their disease and guilt as they felt responsible for their own illness and unable to control their symptoms.^42^
  ‘I know I have to take the responsibility; it's harmful to me if I don't. This is what the nurse said’ (P19 cited in Franklin^37^).
  People with COPD reported an ‘assumed responsibility’^37^; clinicians and family assume that they are able to make informed choices regarding self‐care activities, even though some people report feeling unsure of what types of activity constituted as self‐care and adopted personal strategies on more of a ‘trial and error’ basis.
  Self‐care practices were often self‐initiated as a number of individuals living with COPD understood that they have responsibilities, such as using inhalers correctly, smoking cessation or exercise and attempted to enact these. These enactments are based on personal and peer experiences, experiential constructs fostered by a sense of knowing.^36^ This ‘knowing’ is built on experiences, both personal and from comparing their experiences with others, rather than informed or guided by evidence or teaching and professional support from clinicians.
  ‘When I spoke with my friends and relatives and told them that I had this problem, they taught me a lot and have increased my knowledge’ (Participant 1).^36^
  Self‐care, therefore, is initiated and developed through behaviours based on personal and shared experiences rather than formal support.^35^ Often, peer influences are not considered among individuals with COPD to be self‐care practices, but rather more a set of personal behaviours that enabled people to successfully redesign and manage their day‐to‐day tasks. However, it is acknowledged that this approach often lacked health professional input, based on knowledge derived from experiential constructs that can either positively or negatively influence self‐care adoption and participation. Community and peer interactions offered an inclusivity to self‐care, providing a prolonged sense of independence, even when the disease was progressing.
  ‘They're great. They lift me up on that thing (disability access ramp) because I've got the oxygen’ (P6^39^ in reference to volunteer community transport service).
  However, the failure to perform self‐care practices successfully among people with COPD can lead to an increased sense of self‐blame. Some felt that personal expectations to manage alone were too high and support was not appropriately tailored to their individual needs, causing personal conflicts in adaptive versus taught self‐care practices.^35^ DHT has the potential to combine experiential knowledge with health professional‐directed support. DHT can offer disease‐specific information and advice around specific symptoms, through testimonials, peer and web applications recording and measuring symptoms, reassuring people by finding people with comparable symptoms to build online social support.^33^
2. The value of positive engagements with healthcare professionals for initiating socially supported self‐care.
  In terms of participating in their own care, people with COPD were more willing to engage in consultations when they trusted the healthcare professional and viewed them as investing time in the consultation.^34^, ^36^, ^43^
  Quick or rushed interactions were viewed as a disinvestment of health professionals in the personal aspects and experiences of their condition and therefore lay people were less willing to invest in their own care needs. To motivate participation in self‐care practices, several people with COPD voiced that being personally valued as an individual would enhance their participation and the ethos of a shared self‐care agenda.^33^, ^34^, ^36^, ^37^, ^42^
  ‘The GP would tell me straightaway “This is not on, my friend” I like this GP a lot as I have the opinion that I can talk opening to him about my problems…. that would not happen if I did not trust the doctor’ (P100 cited in Franklin^37^).
  People living with COPD sometimes reported having no recollection of professionally introduced self‐care strategies and little understanding of symptom recognition.^34^ Self‐care participation was viewed as challenging across all studies, requiring trusted and valued relationships with a healthcare professional to successfully incorporate their self‐care participation into disease management.^36^
  Lay health coaching, which involved using lay people as personal life and health supporters, enabled further development of patient–professional relationships by approaching the condition and other complex health needs from the perspective of the person with COPD.
  ‘The other thing is that a health coach can be someone who has medical training but they're not a doctor… they can just sit down and have a cup of coffee and talk about our health’ (Participant 42).
  DHT was also reported to have the potential to optimize the quality of lay professional consultation experiences. It was considered by most people with COPD to be an empowering experience to facilitate conversations and validate complex symptoms. This was related to having the ability to maintain a clear record of symptoms that could be shared real time with the health professional, and therefore, was not just a verbal report of retrospective symptoms, which participants often found difficult to recall. DHT levelled the balance of power in consultations by providing clear documentary evidence of symptoms, focusing discussions for people with COPD to their experiences of their condition.^33^
  ‘A lot of time is wasted in consultations talking about things I don't care about. If I was monitoring here at home then there would be plenty to talk about because the information's collected would be about my COPD, about my symptoms, so that wouldn't be a waste of time, it'd be actually something to talk about and try and figure out, say if it was bad at the time’(P127).^33^
3. Developing a personal understanding of illness through social participation, shared and personal experiences

The perceived physical and emotional struggle with breathlessness is a defining recurrent issue of living with COPD.^35^, ^44^, ^45^ Persistent daily respiratory symptoms limit the ability to engage effectively in self‐care practices due to fear, anxiety and fatigue. In turn, these physical and emotional symptoms require adaptation of tasks (pacing) and the need to change behaviours that trigger symptoms.^35^

To be successful at managing COPD, patients must adapt their life to incorporate daily symptoms of breathlessness and fatigue. They are required to manage breathless, fatigue, cough and wheeze and flareups of these symptoms whilst continuing with

1. household tasks,
2. personal care,
3. social activities,
4. family responsibilities,
5. medical appointments and
6. exercise regimes.^46^

People with COPD reported not associating many of these symptoms with COPD, and preferred a trial and error or learning by experience approach to adapting to their symptoms and self‐care practices.^35^

The shared validation of these symptoms was welcomed and supported through the validation of the fluctuation and worsening of symptoms in discussions with health professionals or DHT.^33^

‘Imagine after my diagnosis I'd be given a device to help me see the differences in a good day of breathing against a bad day of breathing? I know that would have eased the worry I had about every little change I was feeling. I'm sure plenty do panic at the slightest sign of being breathless’ (Patient 132).^33^

DHT, in this sense, can encourage positive help‐seeking and successful feedback from self‐initiated self‐care behaviours among individuals living with COPD. Even here, there are potential personal conflicts in engaging with the self‐care skills taught by healthcare professionals.^35^, ^36^, ^37^, ^40^ People with COPD often seek information about their disease from their peers; through social participation, information is valued to stay abreast of symptoms. However, this may not always be clinically correct.^34^, ^36^, ^38^, ^39^, ^40^

4. Recognizing social network support to guide and validate personal choices in people with COPD

People with COPD preferred to discuss their disease in the context of their social networks in terms of personal narratives related to their condition through stories and experiences.^33^, ^34^, ^36^, ^37^, ^38^, ^39^, ^40^ These personal illness narratives can be both in online and offline social networks and are viewed by people with COPD as valued discussions, providing assurances around symptom perceptions and self‐care strategies through peer feedback.^33^

Social networks consisted of both peer and family members. Familial groups, extended close family networks (particularly in the case of older people, and people from Iran, Pakistan and the Pacific Islands),^34^, ^36^, ^38^ can enable personal choices by providing communication channels to the outside world and providing a conduit for self‐care information.^34^, ^36^, ^37^, ^38^ Close family and positive social structures can support the redistribution of personal and social work, such as cooking and cleaning. Positive relationships within social networks can facilitate discussion around the distribution or adaptation of daily tasks to improve the quality of life of someone with COPD.^34^, ^35^, ^36^, ^37^, ^38^, ^39^

‘People who receive good family support and care from their children get better answers [questionnaire study] are better off than those who live alone; not a hundred percent, but it is less likely. In addition, if [they] do not see or hear well it is not a good relationship with the outside world’ (Participant 16).^36^

The reassignment of emotional and family tasks can lead to social displacement or a loss of their usual social role. In turn, a sense of loss is felt for their social position in the family or wider society (i.e., employment loss).^35^ The church and faith were viewed as having a positive role in the lives of Pacific Islander and Pakistani groups of people with COPD; these were reported to support emotional needs and family needs, and were hugely valued.^34^, ^36^, ^38^

‘All important, spiritual life, if we weren't at Church, I believe we won't be a happy family and be blessed with such obedient children… no one drinks alcohol, no one smokes cigarettes… they will have good futures’ (Tongan man, 81 years^34^).

People living with COPD in the Western communities did not value spiritual social participation^38^ or being part of a spiritual community to the same extent as the Pakistani, Iranian or Pacific Islander communities. However, all studies noted that people with COPD value social support and seek to reduce dependencies when they can.

### 3.2 Third‐order interpretations

Third‐order interpretations are the results of the reinterpretation and translation of the second‐order interpretations, Figure 2. This section can also be termed *synthesis refinement*.^31^

*Experiential knowledge* has formed from working through episodes of illness. Illness experience is formed through personal exposure, having the disease and experiencing the symptoms and witnessing the experiences of illness in people with whom they have close relationships, and their peers.^33^, ^34^, ^35^, ^36^ *Valued practices* are concerned with the practices that people value in their lives, their rituals and activities. In this analysis, these also extend to healthcare professionals and society and the value of people in society beyond illness.^36^, ^37^

People with COPD juggle the looming *inevitability of dependence*, whilst managing the disease and working to retain independence. By seeking, gaining and using experiential knowledge in disease management, people can maintain the balance between dependence and independence.

In these personal experiences with disease, day‐to‐day management (i.e., I carried less shopping) or in acute fluctuations of disease (i.e., I used my inhaled therapy earlier than I usually do and I didn't get so unwell) enables a process of self‐appraisal of disease phenomena. This appraisal draws from narratives or experiences of what has personally worked well in the past. Importantly, ‘working well’ is what has enabled independence, not necessarily what is deemed to be clinically correct disease management. Therefore, if a personal experience is of a peer (friend or relation) dying shortly after stopping smoking, then this is experiential knowledge.^47^ This suggests to people that smoking cessation may contribute to the deterioration of symptoms, lead to the risk of an earlier death and remove a valued activity (smoking). Therefore, people with COPD may dismiss smoking cessation as a viable self‐care option due to their experiential knowledge and the enjoyment they get from smoking, not the advice from a health professional.^33^, ^36^, ^38^

These third‐order interpretative accounts led to three broader overarching concepts that influence both positive and negative engagement along a continuum.

1. Experiential knowledge—Learning from the personal successes and failures of adaptive self‐care behaviours to inform future self‐care practices.
2. Inclusion of valued practices in SSMS.
3. Inevitability of dependence—The fear or avoidance of the inevitability of dependency.

The personal successes achieved from SSMS with adaptive self‐care behaviours can positively inform future self‐care practices and choices. Conversely, negative experiences of social self‐management can reduce self‐efficacy and experiential failures can leave people with a sense of powerlessness, which in turn increases feelings of dependency.

**Figure 3:**
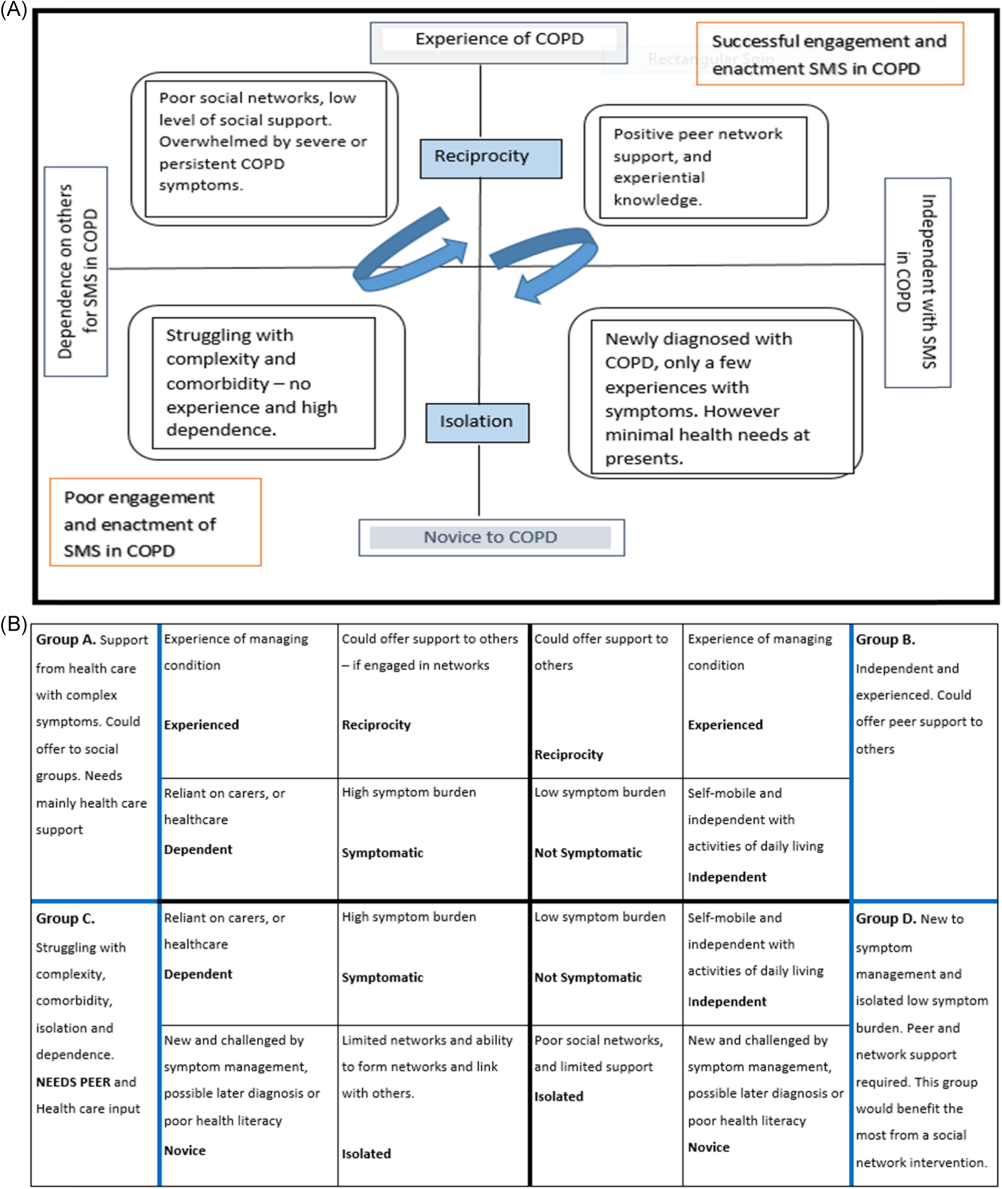
(A) Above: Enactment and engagement of SMS in COPD, the dependence/experience continuum. (B) Below: Adapted conceptual model to depict grouping of people based on independence, experience, symptoms and ability to be reciprocal. COPD, chronic obstructive pulmonary disease

Figure 3A,B shows the Index model that presents the *inde*pendence *ex*perience (Index) continuum. Each person with COPD is either a novice to self‐care or has personal experience based on personal knowledge and previous experience of the disease. Individuals living with COPD move both between novice and experience and their own self‐care enactment, whilst simultaneously striving to remain as independent as they can for as long as they can.

The continuum can be influenced positively through peer support structures and positive social networks, but can be influenced negatively when isolated and a novice in COPD.^48^, ^49^ Furthermore, poor experiences and/or repetitive failures in self‐care or disease complexity can lead to dependency and fear of engagement and enactment in self‐care practices.^33^, ^34^, ^36^, ^40^

### 3.3 Patient and public involvement in the synthesis of the findings

Public collaboration was sought to review the language and accessibility of the manuscript and the real‐world applicability of the findings. The NIHR ARC Wessex long‐term conditions (LTCs) patient and public involvement (PPI) group reviewed the second‐ and third‐order interpretations. The group affirmed the need to address SSMS as relevant aspects of person‐centred care. The group highlighted experiential learning in terms of understanding from their own health needs, in particular, valuing positive trusting relationships with healthcare professionals. A group member (A. A.) volunteered to review the manuscript, paying attention to the accessibility, language and jargon of the manuscript. Specific comments from the PPI group are included in Appendix A.

## 4 DISCUSSION

### 4.1 Main findings

The aim and objectives of this qualitative meta‐ethnography were to focus on participation in social networks to enhance self‐care from the perspective of a person with COPD and to explore the process of adoption of socially supportive self‐care in people with COPD as targets for future self‐care interventions. The findings from this synthesis highlight the complexities involved in social participation linked to self‐care practices in COPD. People with COPD are required to understand and negotiate complex health circumstances and across a range of social contexts. These include the following:

1. day‐to‐day social work‐based negotiations,^16^, ^34^, ^35^, ^36^
2. healthcare interactions,^33^, ^35^, ^36^, ^37^, ^38^, ^39^, ^40^
3. social negotiations concerned with symptom interpretation and^34^, ^35^, ^38^, ^40^
4. acceptance of the disease and its limitations by themselves and others.^33^, ^34^, ^36^, ^37^, ^38^, ^39^, ^40^, ^50^

Therefore, engaging with COPD social networks to enhance self‐care requires multiple skills of negotiation, personal organisation and opportunities to engage in positive peer interactions to be successful.

COPD has a complex, uncertain trajectory, meaning that the pursuit of clinical and social stability is challenging. The longer people live with COPD, the more opportunity there is to enhance the skills and knowledge of self‐care, but these enhanced skills are often against a backdrop of failing health and striving to maintain independence. This study advances COPD self‐care beyond taught healthcare professional interactions towards a more complex interplay with personal and peer‐acquired knowledge and a perceived fear of dependence.

Through developing an understanding of personally valued practices and developing tailored healthcare encounters, one can improve engagement with socially supportive self‐care practices in people with COPD. However, practitioners often prioritize education and information provision over more psychosocial approaches to self‐care.^51^ Russell et al.^12^ undertook a qualitative synthesis of the self‐management literature in people with COPD. Our findings agree with those of Russell et al's.^12^ study, in that healthcare professionals may not have the skills or confidence to address the psychosocial needs of people with COPD, and this in turn limits the capacity for persons with COPD to engage in their own self‐care practices as they do not feel valued. Person‐centred healthcare needs to focus on the person as an individual, placing equal importance on addressing personal issues arising from COPD as well as providing clinical treatment. A narrative synthesis of lay understandings of self‐management in LTCs, including COPD, found that the time spent with a healthcare professional is often interpreted as positive input into self‐care activities.^52^ Healthcare professionals who listen and understand personally valued activities can influence self‐care practices and health outcomes.^36^, ^42^, ^52^ Our findings advance the work of Ambrosio,^53^ who described the process of living with chronic illness, suggesting that successful self‐care practices can only occur once acceptance and coping have been addressed. Integration of new living patterns is applied before being able to live positively with a chronic illness. Our work on lay perspectives of living with COPD advances Ambrosio et al's.^53^ findings and describes the specific issues of dependency and fear, which are heightened in COPD due to persistent and fluctuating breathlessness and fatigue.

Engaging in self‐management activities is positively correlated with the length of time living with the condition^53^ and was supported by the public review of the findings. This aligns with the conceptual model (Figure 3B) in terms of considering the importance of experiential learning in developing a personal and experiential sense of knowing, which is enhanced over time due to living with COPD.^36^ This knowing developed from experiential constructs can, however, both positively and negatively influence participation in SSMS and relies on peer information and a wish on the part of the individual living with COPD to reduce a perceived sense of dependence.^36^, ^37^, ^40^ Prompting the broadening of social networks, as in lay coaching, to support interpretation of symptoms and shared decision making,^40^ can promote independence in people with COPD, enabled through feeling informed to make decisions about their own care, through peer discussion or valued relationships with healthcare professionals.^48^, ^49^

The conceptual model illustrates the independence and experience continuum of engagement in socially supportive self‐care behaviours among people living with COPD. Figure 3A shows the processes of striving for independence, whilst learning self‐care practices and management behaviours improves with experience. Figure 3B maps the social continuum against the intensity of COPD symptoms and reciprocal social behaviours, in this way creating a framework to use in clinical practice to direct social participation in people with COPD. It highlights the personal value of social roles and reciprocal relationships.^24^, ^47^ Sharing ideas and information within peer networks is part of developing a position in a social group. The exchanges and use of peer knowledge are used to maintain independence, in relation to similar others. This independence operates along a dependence and independence continuum and enables us to further our understanding of how people with COPD engage with social support networks and participate in SSMS and self‐care practices.

### 4.2 Implications for future research policy and practice

The conceptual model can be translated into a clinical practice tool. It can be used in clinical consultations to direct social prescribing (linking people to social activities to improve health outcomes) or social support decisions in partnership with healthcare professionals. The Index model could serve to provide a framework to structure patient–healthcare professional interactions^12^ and facilitate conversations on the role of social support and networks in supporting SSMS and self‐care practices among people with COPD.

Healthcare professionals should consider integrating peer support and lay coaching into existing healthcare pathways to encourage purposefully selected peer encounters to enable supportive, positive self‐care practices. Current work to test the conceptual model with healthcare professionals is in progress.

### 4.3 Strengths and limitations

This qualitative meta‐ethnography has strength in terms of the scrutiny of the methodology, drawn from several sources,^26^, ^27^, ^31^ and review of the analysis by an interdisciplinary team of researchers. This oversight of the formulation of the concepts confers validity to the conceptual models. Further review of the findings was carried out by a PPI representative as an editor of this manuscript. The meta‐ethnography is limited by the small number of studies included, although these were robustly selected.

## 5 CONCLUSION

This study provides insights into how and why people engage with the principles and practices of socially supportive self‐care and the role of participation in social networks in supporting self‐care activities and practices from the perspective of people living with COPD. Through mapping the findings of the social continuum with the intensity of COPD symptoms, the Index model has been developed for use in clinical practice to measure the social capacity and direct social participation in people with COPD.

The conceptual model can (i) explain how and why people with COPD understand and engage with social network support to manage their COPD and (ii) provide guidance for healthcare professionals to engage in person‐centred conversations relating to valued activities and personal care preferences.

Socially supportive self‐care warrants embracing as a fundamental element an encouraging infrastructure of people to assistant in the lives of the person with COPD. In the long term, this can lead to the development of positive cognitive, emotional and physical self‐care practices to continually improve health outcomes in people with COPD.

## CONFLICT OF INTERESTS

The authors declare that there are no conflict of interests.

## AUTHOR CONTRIBUTIONS

Lindsay Welch designed and delivered the qualitative meta‐ethnography, undertook quality appraisal, interpreted the findings, conceptualized the tool and drafted the manuscript. Euan Sadler read the papers, contributed to the analysis and synthesis and reviewed and edited the manuscript for publication. Anne Rogers conceptualized the work, advised on the design process, reviewed and quality‐appraised the selected studies and reviewed and edited the manuscript for publication. Anthony Austin reviewed and edited the manuscript from the perspective of a lay user. Anthony reviewed the language and the findings, and commented on the relevance of the findings to user experiences. All the listed authors have read and approved the manuscript.

## Data Availability

The data that support the findings of this study are available in the Supporting Information Material of this article.

## APPENDIX A

Comments from the patient and public involvement (PPI) group meeting on 10 December 2020. Permission was obtained from the members of the PPI groups to share their verbatim comments. The findings of the study were presented in diagrammatic form; the group was asked three questions. Each question was discussed in turn. These were the comments from the feedback and the discussion.

**Question 1:** Do these findings feel ‘real’—can you relate them to how people with long term conditions my feel?

**Question 2:** Do you think they are a fair representation of people with a long‐term respiratory condition?

**Comments**

‘Very similar to work on IBD—SM is important, and the collaboration aspect is key—you need to be able to build a relationship with your health professional to make it work’ (PPI contributor 1).

‘Self‐management needs to be INFORMED self‐management, it only works if you are informed and understand how to manage yourself. Especially when an adolescent, your views on your illness and your ability to manage your illness differ with age’ (PPI contributor 2).

‘Having clinicians open to discuss SMS and appreciate patient experiences is important. We are living with the disease. Without the creation of a two‐way challenge and dialogue we are likely to fail at SM. We need reciprocal relationships with healthcare’ (PPI contributor 3).

*‘*As a post‐transplant patient, I will always be a patient, so therefore have a long‐term condition. I benefit from shared experiences and groups with other transplant patients. Debates and decisions on care, vaccination with a compromised immune system’ (PPI contributor 4).

**Question 3:** Is there a point of finding that you are uncertain about—or does not fit with your thinking and you would like to discuss?

**Comments**

‘Perhaps you are not reflecting access to support at crisis points, and when people are alone. This is the day to day management, discussing the differences when a person is in a crisis—such as a respiratory crisis is important’ (PPI contributor 2).

‘Anxiety and breathlessness do draw and the work on asthma research’ (PPI contributor 5).

‘Mention the importance of technological advancements and collaboration with health care and digital’ (PPI contributor 1).

The PPI group broadly concur with the findings and the application of the findings in people with long‐term conditions and in real‐world scenarios. Crisis action plans do have a place and the social network approach could be used to support crisis decision making.

However, more work needs to be completed to understand the role of socially supported self‐management at the point of crisis decision making. The PPI group were overall supportive of the conceptual model and particularly valued the core aspects of involvement in care and treatment planning and the inclusion of social or peer influences *(PPI contributors 1, 2, 3)*. Engaging clinical teams in discussing and implementing ‘valued activities and a more psychosocial approach to SMS’ resonated with the group, therefore this is something to be considered as part of routine care in people with COPD and LTC.
